# Effects of Interobserver Segmentation Variability and Intensity Discretization on MRI-Based Radiomic Feature Reproducibility of Lipoma and Atypical Lipomatous Tumor

**DOI:** 10.1007/s10278-024-00999-x

**Published:** 2024-02-08

**Authors:** Salvatore Gitto, Renato Cuocolo, Vincenzo Giannetta, Julietta Badalyan, Filippo Di Luca, Stefano Fusco, Giulia Zantonelli, Domenico Albano, Carmelo Messina, Luca Maria Sconfienza

**Affiliations:** 1 IRCCS Istituto Ortopedico Galeazzi, Via Cristina Belgioioso 173, 20157 Milan, Italy; 2 Dipartimento Di Scienze Biomediche Per La Salute, Università Degli Studi Di Milano, Milan, Italy; 3 Department of Medicine, Surgery and Dentistry, University of Salerno, Baronissi, Italy; 4 Diagnostic and Interventional Radiology Department, IRCCS Ospedale San Raffaele-Turro, Università Vita-Salute San Raffaele, Milan, Italy; 5 Scuola Di Specializzazione in Statistica Sanitaria E Biometria, Università Degli Studi Di Milano, Milan, Italy; 6 Scuola Di Specializzazione in Radiodiagnostica, Università Degli Studi Di Milano, Milan, Italy; 7 Dipartimento Di Scienze Biomediche, Chirurgiche Ed Odontoiatriche, Università Degli Studi Di Milano, Milan, Italy

**Keywords:** Artificial intelligence, Atypical lipomatous tumor, Lipoma, Radiomics, Texture analysis

## Abstract

Segmentation and image intensity discretization impact on radiomics workflow. The aim of this study is to investigate the influence of interobserver segmentation variability and intensity discretization methods on the reproducibility of MRI-based radiomic features in lipoma and atypical lipomatous tumor (ALT). Thirty patients with lipoma or ALT were retrospectively included. Three readers independently performed manual contour-focused segmentation on T1-weighted and T2-weighted sequences, including the whole tumor volume. Additionally, a marginal erosion was applied to segmentations to evaluate its influence on feature reproducibility. After image pre-processing, with included intensity discretization employing both fixed bin number and width approaches, 1106 radiomic features were extracted from each sequence. Intraclass correlation coefficient (ICC) 95% confidence interval lower bound ≥ 0.75 defined feature stability. In contour-focused vs. margin shrinkage segmentation, the rates of stable features extracted from T1-weighted and T2-weighted images ranged from 92.68 to 95.21% vs. 90.69 to 95.66% after fixed bin number discretization and from 95.75 to 97.65% vs. 95.39 to 96.47% after fixed bin width discretization, respectively, with no difference between the two segmentation approaches (*p* ≥ 0.175). Higher stable feature rates and higher feature ICC values were found when implementing discretization with fixed bin width compared to fixed bin number, regardless of the segmentation approach (*p* < 0.001). In conclusion, MRI radiomic features of lipoma and ALT are reproducible regardless of the segmentation approach and intensity discretization method, although a certain degree of interobserver variability highlights the need for a preliminary reliability analysis in future studies.

**Supplementary Information:**

The online version contains supplementary material available at 10.1007/s10278-024-00999-x.

## Introduction

Atypical lipomatous tumor (ALT) and lipoma are the most common soft-tissue lesions [1]. According to the 2020 edition of the World Health Organization classification [2], the term ALT is reserved for low-grade adipocytic neoplasms arising at anatomical sites for which surgery is generally curative, including the extremities and trunk [2]. ALTs have a relatively indolent disease course compared to well-differentiated liposarcomas, namely lipomatous lesions with the same histology but located in deep anatomical sites such as the retroperitoneum, mediastinum, and spermatic cord, where there is a higher risk for recurrence and dedifferentiation related to lower chances of achieving negative surgical margins [2]. In line with this relatively indolent clinical behavior, treatment strategy has progressively shifted from extensive surgery to marginal excision in ALTs, which is now considered an appropriate option to achieve local control while taking into account the morbidity rates associated with surgery [3]. On the other hand, lipomas are benign lipomatous lesions, which do not require any treatment unless symptomatic or due to cosmetic concerns [3]. Lipomas are rare in deep locations, such as the retroperitoneum, but very common in the extremities and trunk [1]. Thus, an accurate distinction between ALT and lipoma is desirable to offer optimal patient care.

In the diagnostic pathway of lipomatous soft-tissue lesions, magnetic resonance imaging (MRI) is the imaging method of choice for diagnosis and differentiating ALT from lipoma, with high sensitivity and substantial specificity [4–6]. In detail, according to a recent meta-analysis, the sensitivity and specificity of radiologists evaluating multiple combined imaging parameters (called “radiologist gestalt”) range from 76 to 100% and 37 to 77%, respectively, if only studies focusing on lipoma and ALT are considered [4]. Nonetheless, a certain degree of interobserver variability has emerged even among expert readers [5–7], with kappa values ranging from 0.23 to 0.7 according to this meta-analysis [4]. Preliminary imaging studies applying radiomics have shown promise for improving diagnostic accuracy and characterizing lipomatous soft-tissue lesions more objectively [8]. Radiomics includes the extraction and analysis of quantitative parameters from medical images, known as radiomic features [9–11]. A crucial step of radiomic workflows is feature reproducibility assessment, as these quantitative parameters may suffer from interobserver variability, particularly regarding tumor delineation while performing manual segmentation [12–15]. Segmentation margins are also critical because the peritumoral area may influence the reproducibility of radiomic features and their diagnostic performance [15, 16]. Furthermore, in radiomic workflows, the effects of different image intensity discretization methods on feature reproducibility are debated [17–19]. In literature, the intraclass correlation coefficient (ICC) is commonly employed to evaluate radiomic feature reproducibility [16, 20–23].

The aim of this study is to investigate the influence of interobserver manual segmentation variability on the reproducibility of MRI-based radiomic features in lipoma and ALT, also considering the impact of different image intensity discretization methods.

## Materials and Methods

### Design and Population

Institutional Review Board approved this retrospective study and waived the need for informed consent. This study was designed to meet the numerical requirements of a reproducibility analysis in terms of patients and readers involved, namely 30 lesions and 3 different readers, according to the ICC guidelines by Koo and Li [24]. An electronic search of the pathology information system was performed, and 30 patients with lipomatous soft-tissue tumors were included (median age 58 [range 40–79] years). Inclusion criteria were as follows: (i) lipoma or ALT proven by post-surgical pathology, which was based on microscopic findings and MDM2 immunohistochemistry or fluorescence in situ hybridization; (ii) 1.5-T MRI performed within 3 months before surgery, including turbo spin echo T1-weighted and T2-weighted sequences without fat suppression. Exclusion criteria were ALT local recurrence and poor image quality or image artifacts affecting segmentation and radiomic analysis.

Details regarding location, size, and main imaging characteristics of the included lipomas and ALTs are provided in Table 1. All examinations were performed on one of two 1.5-T MRI systems (Magnetom Avanto or Magnetom Espree, Siemens Healthineers, Erlangen, Germany). Axial T1-weighted and T2-weighted MRI sequences were extracted for image analysis. The median matrix size and slice thickness were 512 × 512 (range 320–512 × 216–512) and 3.5 (range 3–5) mm, respectively. The median TE and TR were 11 (range 10–21) and 663 (range 454–800) ms on T1-weighted sequences, respectively. The median TE and TR were 100 (range 80–146) and 3664 (range 2000–7444) ms on T2-weighted sequences, respectively. All extracted DICOM images were converted to the NiFTI format prior to the analysis.

**Table 1:** Location, size and main imaging characteristics of the ALTs and lipomas included in this study

	**ALT**	**Lipoma**
Anatomical site	Arm, *n* = 2 Forearm or hand, *n* = 4 Leg, *n* = 1 Thigh, *n* = 9	Arm, *n* = 4 Forearm or hand, *n* = 5 Leg, *n* = 1 Thigh, *n* = 4
Location relative to fascia	All deep to the deep peripheral fascia surrounding muscles	All deep to the deep peripheral fascia surrounding muscles
Maximum diameter	145 (43–292) mm	83 (32–155) mm
Thick septations (> 2 mm)	Yes, *n* = 10 No, *n* = 6	Yes, *n* = 6 No, *n* = 8
Non-fatty nodular/irregular components	Yes, *n* = 1 No, *n* = 15	Yes, *n* = 0 No, *n* = 14

Maximum diameter is expressed as median (range)

### Image Segmentation

**Fig. 1:**
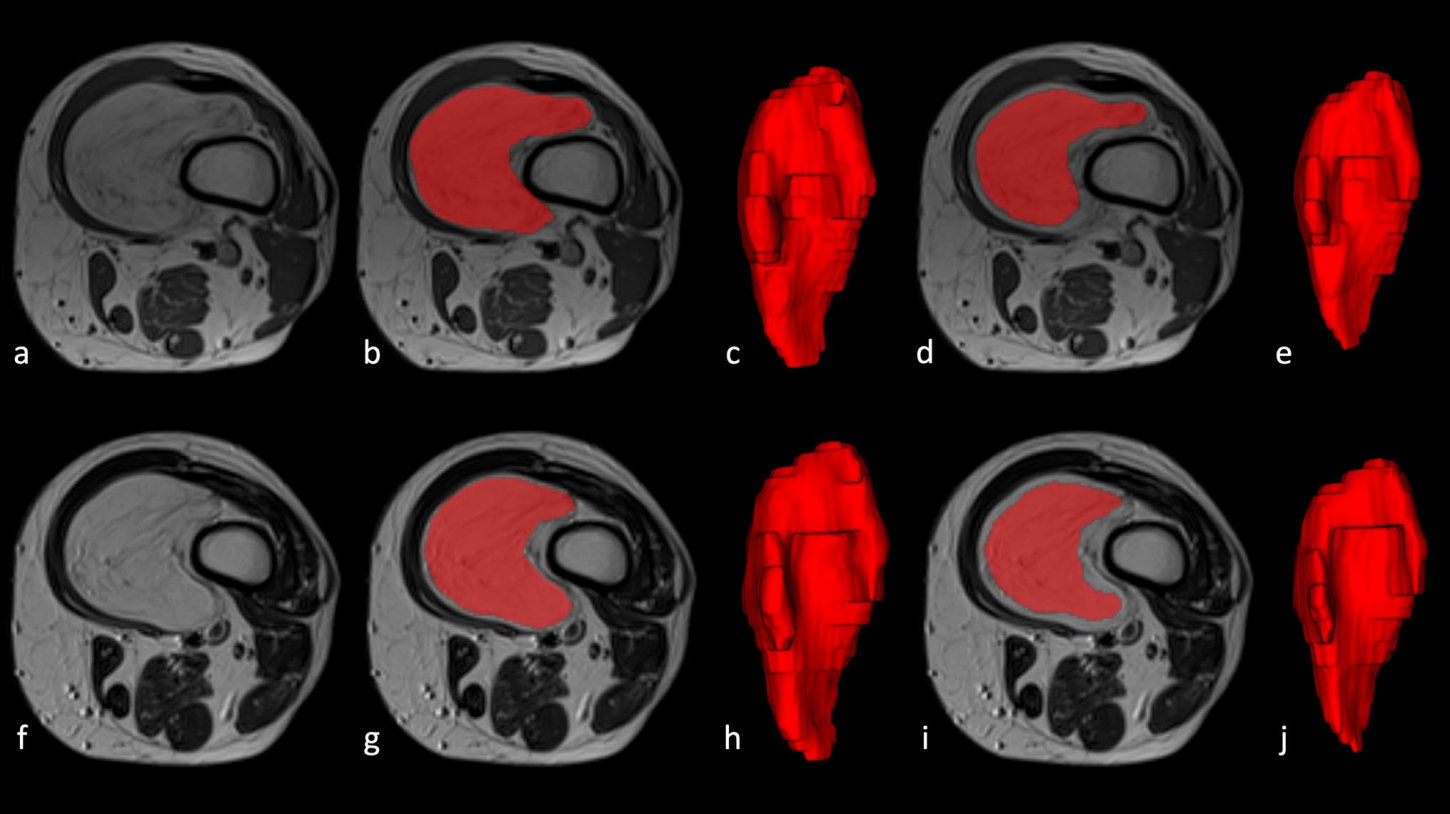
The upper and lower rows present two different examples of lesion annotation. These include the original images (**a**, **f**) with corresponding contour-focused segmentation presented as **a** mask (**b**, **g**) and relative 3D volume (**c**, **h**). Finally, the results of automated margin shrinkage are shown for both the mask (**d**, **i**) and volume (**e**, **j**)

A musculoskeletal radiologist with 4 years of experience in musculoskeletal tumor imaging (S.G.), a general radiologist (V.G.), and a medical resident (J.B.) independently performed manual image segmentation using the open-source software ITK-SNAP (v3.8) [25]. The readers knew the study would deal with lipomatous soft-tissue tumors, but they were blinded to any additional information regarding pathology or disease course. Manual contour-focused segmentation was performed by drawing a region of interest (ROI) slice by slice to include the whole tumor volume on both axial T1-weighted and T2-weighted MRI sequences. Thereafter, margin shrinkage segmentation was computed by applying a marginal erosion to evaluate the influence of segmentation margins on feature reproducibility (Fig. 1). In detail, ROI shrinkage was performed using the fslmaths erosion function of the FMRIB Software Library [26]. The default kernels, namely a 3 × 3 × 3 box centered at the target voxel, were employed.

### Radiomic Analysis

Image pre-processing and feature extraction were performed using PyRadiomics (v3.0.1) [27], an open-source Python software. Image pre-processing consisted of resampling to a 2 × 2 × 2 isotropic voxel, intensity normalization (mean value of 300 and standard deviation of 100) and discretization with both options of fixed bin number and fixed bin width, as implemented in PyRadiomics. In detail, discretization was obtained using both a fixed bin number of 64 and a fixed bin width of 7.

Original images were used for extraction of first-order, shape-based and texture features, which were grouped according to PyRadiomics official documentation (https://pyradiomics.readthedocs.io/en/latest/features.html) and included: 18 first-order features, 14 shape-based features, 22 Gy-level cooccurrence matrix (GLCM) features, 16 Gy-level size zone matrix (GLSZM) features, 16 Gy-level run length matrix (GLRLM) features, 14 Gy-level dependence matrix (GLDM) features, and 5 neighboring gray tone difference matrix (NGTDM).

In addition to the original images, Laplacian of Gaussian (LoG)–filtered (sigma = 2, 4, 6) and wavelet-transformed images (all possible low and high pass filter combinations) were obtained for extraction of first-order and texture features. Shape-based features are independent from gray-level value distribution and therefore were only computed on the original images. A total of 1106 features were extracted from original, LoG-filtered, and wavelet-transformed images for each MRI sequence.

### Statistical Analysis

Interobserver reliability was assessed using two-way, random-effects, single-rater agreement ICC 95% confidence interval (CI) lower bound. Features were considered stable when achieving good (0.75 ≤ ICC 95% CI lower bound < 0.9) to excellent (ICC 95% CI lower bound ≥ 0.9) interobserver reliability [24]. Differences among stable feature rates were evaluated using chi-square test. Differences among ICC 95% CI lower bound values were evaluated using Friedman test for repeated samples and Wilcoxon signed rank test with continuity correction for pairwise comparisons. A two-sided *p*-value < 0.05 indicated statistical significance [28]. Data analysis was performed using the pandas and numpy Python software and the “irr” R package [29, 30].

## Results

### Stable Feature Rates by Intensity Discretization Method and Segmentation Approach

**Fig. 2:**
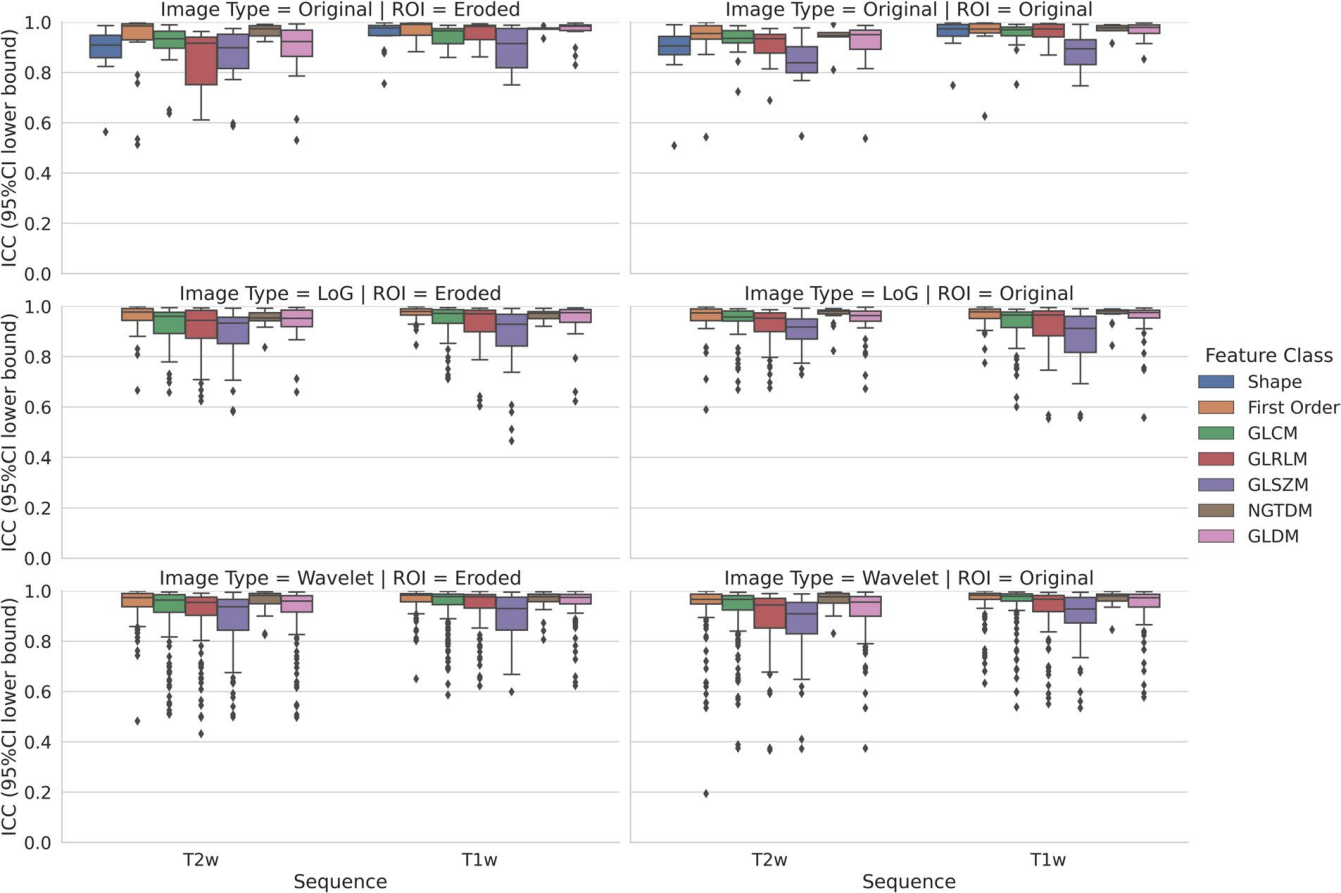
Contour-focused (original ROI) vs. margin shrinkage (eroded ROI) segmentation after image intensity discretization with fixed bin number. Box and whisker plots show the interobserver reproducibility of feature classes grouped according to image type and MRI sequence. GLCM, gray-level cooccurrence matrix; GLDM, gray-level dependence matrix; GLRLM, gray-level run length matrix; GLSZM, gray-level size zone matrix; ICC, intraclass correlation coefficient; LoG, Laplacian of Gaussian; NGTDM, neighboring gray tone difference matrix

After implementing image intensity discretization with fixed bin number, in contour-focused vs. margin shrinkage segmentation, the stable feature rates were 95.21% (*n* = 1053) vs. 95.66% (*n* = 1058) and 92.68% (*n* = 1025) vs. 90.69% (*n* = 1003) for T1-weighted and T2-weighted images, respectively, with no statistical difference (*p* = 0.298). In Fig. 2, box and whisker plots show the interobserver reproducibility of feature classes derived from contour-focused and margin shrinkage segmentations, grouped according to image type and MRI sequence. The matching stable features derived from contour-focused and margin shrinkage segmentations performed on T1-weighted and T2-weighted images were 92.68% (*n* = 1025) and 86.80% (*n* = 960), respectively, as detailed in Supplementary Files 1–2.

**Fig. 3:**
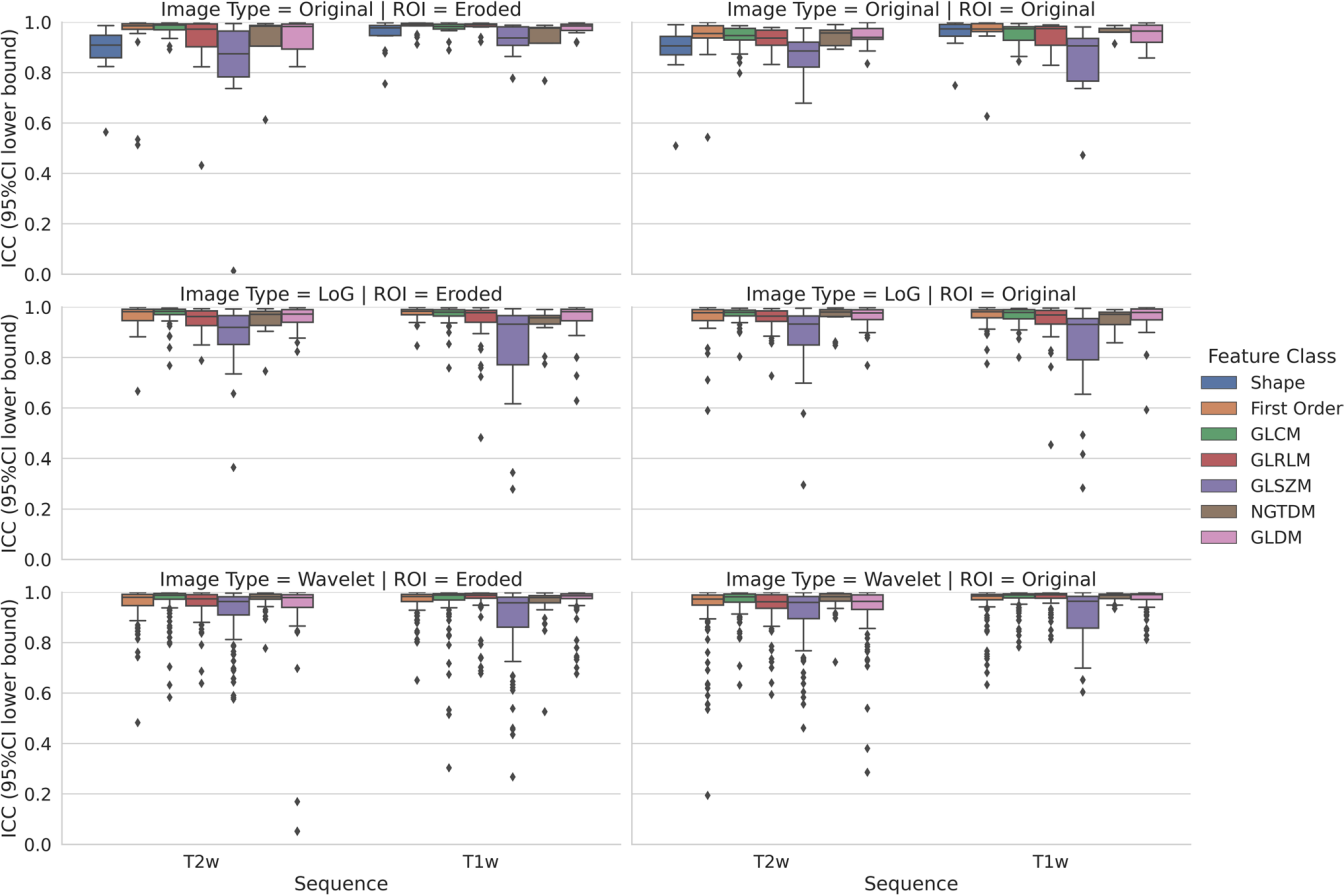
Contour-focused (original ROI) vs. margin shrinkage (eroded ROI) segmentation after image intensity discretization with fixed bin width. Box and whisker plots show the interobserver reproducibility of feature classes grouped according to image type and MRI sequence. GLCM, gray-level cooccurrence matrix; GLDM, gray-level dependence matrix; GLRLM, gray-level run length matrix; GLSZM, gray-level size zone matrix; ICC, intraclass correlation coefficient; LoG, Laplacian of Gaussian; NGTDM, neighboring gray tone difference matrix

After implementing image intensity discretization with fixed bin width, in contour-focused vs. margin shrinkage segmentation, the stable feature rates were 97.65% (*n* = 1080) vs. 95.39% (*n* = 1055) and 95.75% (*n* = 1059) vs. 96.47% (*n* = 1067) for T1-weighted and T2-weighted images, respectively, with no statistical difference (*p* = 0.175). In Fig. 3, box and whisker plots show the interobserver reproducibility of feature classes derived from contour-focused and margin shrinkage segmentations, grouped according to image type and MRI sequence. The matching stable features derived from contour-focused and margin shrinkage segmentations performed on T1- and T2-weighted images were 94.30% (*n* = 1043) and 93.76% (*n* = 1037), respectively, as detailed in Supplementary Files 3–4.

In image intensity discretization with fixed bin number vs. fixed bin width, the latter discretization method yielded higher rates of stable features regardless of the segmentation approach (*p* < 0.001). Tables 2, 3, 4 and 5 show the number and percentage of stable features that were obtained with different combinations of discretization methods and segmentation approaches, grouped according to feature class and image type.

**Table 2:** Discretization with fixed bin number and contour-focused segmentation. Number and percentage of stable features with good (0.75 ≤ ICC 95% CI lower bound < 0.9) and excellent (ICC 95% CI lower bound ≥ 0.9) interobserver reproducibility grouped according to feature class and image type

**Image**	**Feature class**	**Image type**	**Total feature number (*n*)**	**ICC ≥ 0.75 (*n*)**	**ICC ≥ 0.90 (*n*)**	**ICC ≥ 0.75 (%)**	**ICC ≥ 0.90 (%)**
**T1w**	**First order**	LoG	54	54	50	100	92.59
		Original	18	17	17	94.44	94.44
		Wavelet	144	138	130	95.83	90.28
	**Shape**	Original	14	13	13	92.86	92.86
	**GLCM**	LoG	66	63	51	95.45	77.27
		Original	22	22	20	100	90.91
		Wavelet	176	166	157	94.32	89.2
	**GLDM**	LoG	42	40	36	95.24	85.71
		Original	14	14	13	100	92.86
		Wavelet	112	107	98	95.54	87.5
	**GLRLM**	LoG	48	45	34	93.75	70.83
		Original	16	16	15	100	93.75
		Wavelet	128	120	104	93.75	81.25
	**GLSZM**	LoG	48	43	26	89.58	54.17
		Original	16	15	7	93.75	43.75
		Wavelet	128	120	77	93.75	60.16
	**NGTDM**	LoG	15	15	14	100	93.33
		Original	5	5	5	100	100
		Wavelet	40	40	39	100	97.5
	**Overall**		**1106**	**1053**	**906**	**95.21**	**81.92**
**T2w**	**First order**	LoG	54	52	49	96.3	90.74
		Original	18	17	15	94.44	83.33
		Wavelet	144	134	123	93.06	85.42
	**Shape**	Original	14	13	8	92.86	57.14
	**GLCM**	LoG	66	63	56	95.45	84.85
		Original	22	21	18	95.45	81.82
		Wavelet	176	162	143	92.05	81.25
	**GLDM**	LoG	42	40	36	95.24	85.71
		Original	14	13	10	92.86	71.43
		Wavelet	112	106	84	94.64	75
	**GLRLM**	LoG	48	45	36	93.75	75
		Original	16	15	10	93.75	62.5
		Wavelet	128	114	87	89.06	67.97
	**GLSZM**	LoG	48	47	29	97.92	60.42
		Original	16	15	4	93.75	25
		Wavelet	128	108	67	84.38	52.34
	**NGTDM**	LoG	15	15	14	100	93.33
		Original	5	5	4	100	80
		Wavelet	40	40	38	100	95
	**Overall**		**1106**	**1025**	**831**	**92.68**	**75.14**

*GLCM* gray-level cooccurrence matrix, *GLDM* gray-level dependence matrix, *GLRLM* gray-level run length matrix, *GLSZM* gray-level size zone matrix, *ICC* intraclass correlation coefficient, *LoG* Laplacian of Gaussian, *NGTDM* neighboring gray tone difference matrix

**Table 3:** Discretization with fixed bin number and margin shrinkage segmentation. Number and percentage of stable features with good (0.75 ≤ ICC 95% CI lower bound < 0.9) and excellent (ICC 95% CI lower bound ≥ 0.9) interobserver reproducibility grouped according to feature class and image type

**Image**	**Feature class**	**Image type**	**Total feature number (*n*)**	**ICC ≥ 0.75 (*n*)**	**ICC ≥ 0.90 (*n*)**	**ICC ≥ 0.75 (%)**	**ICC ≥ 0.90 (%)**
**T1w**	**First order**	LoG	54	54	53	100	98.15
		Original	18	18	17	100	94.44
		Wavelet	144	143	132	99.31	91.67
	**Shape**	Original	14	14	11	100	78.57
	**GLCM**	LoG	66	63	52	95.45	78.79
		Original	22	22	18	100	81.82
		Wavelet	176	166	143	94.32	81.25
	**GLDM**	LoG	42	40	37	95.24	88.1
		Original	14	14	11	100	78.57
		Wavelet	112	108	96	96.43	85.71
	**GLRLM**	LoG	48	44	34	91.67	70.83
		Original	16	16	13	100	81.25
		Wavelet	128	121	104	94.53	81.25
	**GLSZM**	LoG	48	43	28	89.58	58.33
		Original	16	16	9	100	56.25
		Wavelet	128	116	75	90.63	58.59
	**NGTDM**	LoG	15	15	15	100	100
		Original	5	5	5	100	100
		Wavelet	40	40	37	100	92.5
	**Overall**		**1106**	**1058**	**890**	**95.66**	**80.47**
**T2w**	**First order**	LoG	54	53	48	98.15	88.89
		Original	18	16	14	88.89	77.78
		Wavelet	144	142	123	98.61	85.42
	**Shape**	Original	14	13	7	92.86	50
	**GLCM**	LoG	66	62	49	93.94	74.24
		Original	22	20	14	90.91	63.64
		Wavelet	176	157	139	89.2	78.98
	**GLDM**	LoG	42	39	37	92.86	88.1
		Original	14	12	10	85.71	71.43
		Wavelet	112	99	88	88.39	78.57
	**GLRLM**	LoG	48	41	32	85.42	66.67
		Original	16	12	9	75	56.25
		Wavelet	128	110	98	85.94	76.56
	**GLSZM**	LoG	48	41	31	85.42	64.58
		Original	16	14	8	87.5	50
		Wavelet	128	112	82	87.5	64.06
	**NGTDM**	LoG	15	15	14	100	93.33
		Original	5	5	5	100	100
		Wavelet	40	40	37	100	92.5
	**Overall**		**1106**	**1003**	**845**	**90.69**	**76.4**

*GLCM* gray-level cooccurrence matrix, *GLDM* gray-level dependence matrix, *GLRLM* gray-level run length matrix, *GLSZM*, gray-level size zone matrix, *ICC* intraclass correlation coefficient, *LoG* Laplacian of Gaussian, *NGTDM* neighboring gray tone difference matrix

**Table 4:** Discretization with fixed bin width and contour-focused segmentation. Number and percentage of stable features with good (0.75 ≤ ICC 95% CI lower bound < 0.9) and excellent (ICC 95% CI lower bound ≥ 0.9) interobserver reproducibility grouped according to feature class and image type

**Image**	**Feature class**	**Image type**	**Total feature number (*n*)**	**ICC ≥ 0.75 (*n*)**	**ICC ≥ 0.90 (*n*)**	**ICC ≥ 0.75 (%)**	**ICC ≥ 0.90 (%)**
**T1w**	**First order**	LoG	54	54	50	100	92.59
		Original	18	17	17	94.44	94.44
		Wavelet	144	138	130	95.83	90.28
	**Shape**	Original	14	13	13	92.86	92.86
	**GLCM**	LoG	66	66	63	100	95.45
		Original	22	22	19	100	86.36
		Wavelet	176	176	169	100	96.02
	**GLDM**	LoG	42	41	38	97.62	90.48
		Original	14	14	12	100	85.71
		Wavelet	112	112	106	100	94.64
	**GLRLM**	LoG	48	47	43	97.92	89.58
		Original	16	16	14	100	87.5
		Wavelet	128	128	121	100	94.53
	**GLSZM**	LoG	48	39	32	81.25	66.67
		Original	16	13	8	81.25	50
		Wavelet	128	124	93	96.88	72.66
	**NGTDM**	LoG	15	15	12	100	80
		Original	5	5	5	100	100
		Wavelet	40	40	40	100	100
	**Overall**		**1106**	**1080**	**985**	**97.65**	**89.06**
**T2w**	**First order**	LoG	54	52	49	96.3	90.74
		Original	18	17	15	94.44	83.33
		Wavelet	144	134	123	93.06	85.42
	**Shape**	Original	14	13	8	92.86	57.14
	**GLCM**	LoG	66	66	64	100	96.97
		Original	22	22	17	100	77.27
		Wavelet	176	174	164	98.86	93.18
	**GLDM**	LoG	42	42	38	100	90.48
		Original	14	14	12	100	85.71
		Wavelet	112	106	92	94.64	82.14
	**GLRLM**	LoG	48	47	43	97.92	89.58
		Original	16	16	12	100	75
		Wavelet	128	123	113	96.09	88.28
	**GLSZM**	LoG	48	44	31	91.67	64.58
		Original	16	15	6	93.75	37.5
		Wavelet	128	115	94	89.84	73.44
	**NGTDM**	LoG	15	15	12	100	80
		Original	5	5	4	100	80
		Wavelet	40	39	38	97.5	95
	**Overall**		**1106**	**1059**	**935**	**95.75**	**84.54**

*GLCM* gray-level cooccurrence matrix, *GLDM* gray-level dependence matrix, *GLRLM* gray-level run length matrix, *GLSZM* gray-level size zone matrix, *ICC* intraclass correlation coefficient, *LoG* Laplacian of Gaussian, *NGTDM* neighboring gray tone difference matrix

**Table 5:** Discretization with fixed bin width and margin shrinkage segmentation. Number and percentage of stable features with good (0.75 ≤ ICC 95% CI lower bound < 0.9) and excellent (ICC 95% CI lower bound ≥ 0.9) interobserver reproducibility grouped according to feature class and image type

**Image**	**Feature class**	**Image type**	**Total feature number (*n*)**	**ICC ≥ 0.75 (*n*)**	**ICC ≥ 0.90 (*n*)**	**ICC ≥ 0.75 (%)**	**ICC ≥ 0.90 (%)**
**T1w**	**First order**	LoG	54	54	53	100	98.15
		Original	18	18	18	100	100
		Wavelet	144	143	132	99.31	91.67
	**Shape**	Original	14	14	11	100	78.57
	**GLCM**	LoG	66	66	63	100	95.45
		Original	22	22	21	100	95.45
		Wavelet	176	171	165	97.16	93.75
	**GLDM**	LoG	42	40	36	95.24	85.71
		Original	14	14	14	100	100
		Wavelet	112	107	104	95.54	92.86
	**GLRLM**	LoG	48	46	41	95.83	85.42
		Original	16	16	16	100	100
		Wavelet	128	121	119	94.53	92.97
	**GLSZM**	LoG	48	38	30	79.17	62.5
		Original	16	16	12	100	75
		Wavelet	128	110	93	85.94	72.66
	**NGTDM**	LoG	15	15	13	100	86.67
		Original	5	5	4	100	80
		Wavelet	40	39	35	97.5	87.5
	**Overall**		**1106**	**1055**	**980**	**95.39**	**88.61**
**T2w**	**First order**	LoG	54	53	51	98.15	94.44
		Original	18	16	16	88.89	88.89
		Wavelet	144	142	125	98.61	86.81
	**Shape**	Original	14	13	7	92.86	50
	**GLCM**	LoG	66	66	62	100	93.94
		Original	22	22	21	100	95.45
		Wavelet	176	173	165	98.3	93.75
	**GLDM**	LoG	42	42	39	100	92.86
		Original	14	13	10	92.86	71.43
		Wavelet	112	108	93	96.43	83.04
	**GLRLM**	LoG	48	48	43	100	89.58
		Original	16	15	13	93.75	81.25
		Wavelet	128	126	111	98.44	86.72
	**GLSZM**	LoG	48	45	33	93.75	68.75
		Original	16	14	7	87.5	43.75
		Wavelet	128	113	97	88.28	75.78
	**NGTDM**	LoG	15	14	14	93.33	93.33
		Original	5	4	4	80	80
		Wavelet	40	40	38	100	95
	**Overall**		**1106**	**1067**	**949**	**96.47**	**85.8**

*GLCM* gray-level cooccurrence matrix, *GLDM* gray-level dependence matrix, *GLRLM* gray-level run length matrix, *GLSZM* gray-level size zone matrix, *ICC* intraclass correlation coefficient, *LoG* Laplacian of Gaussian, *NGTDM* neighboring gray tone difference matrix

### Feature ICC Values by Intensity Discretization Method and Segmentation Approach

**Fig. 4:**
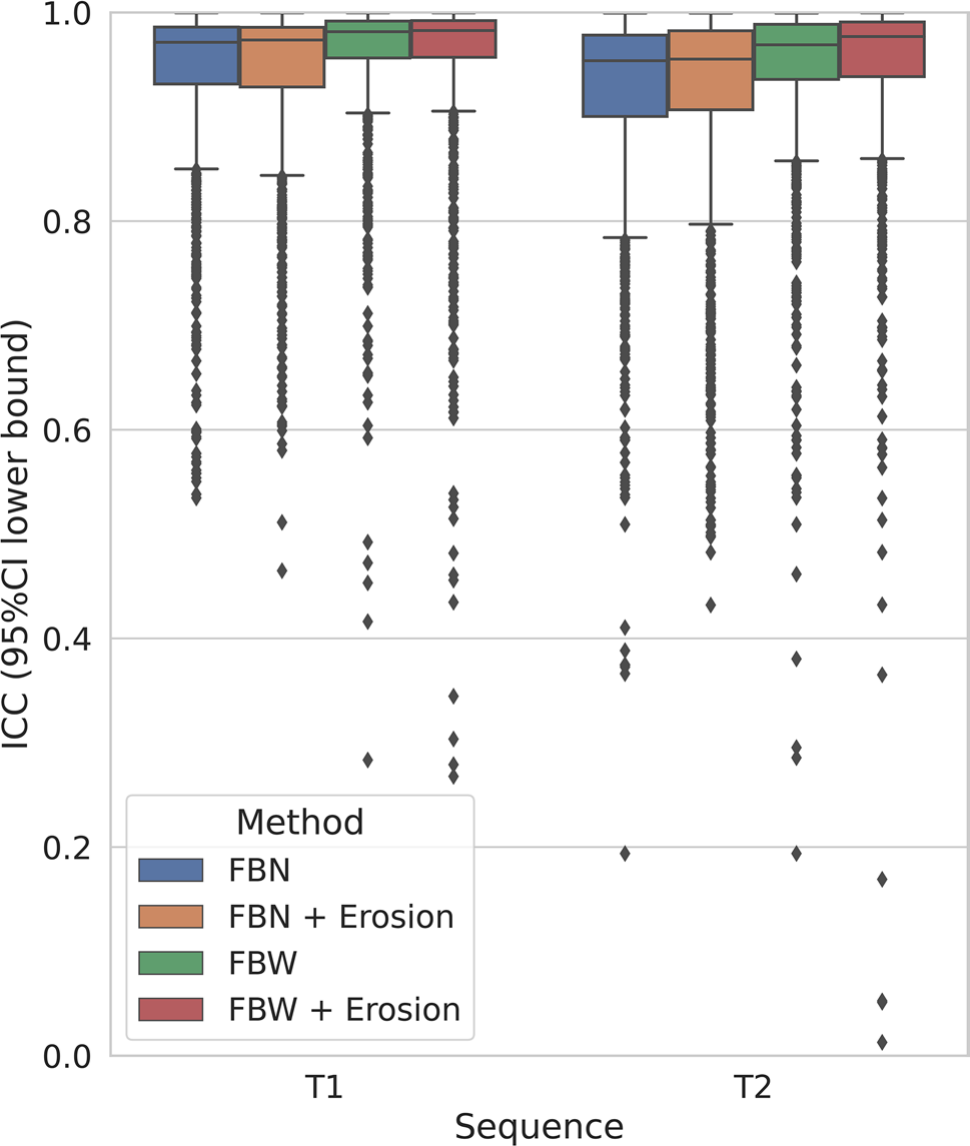
Interobserver reproducibility by discretization method and segmentation approach. Box and whisker plots show the interobserver reproducibility of all features extracted using different discretization methods and ROIs without (contour focused segmentation) or with marginal erosion (margin shrinkage segmentation), grouped according to MRI sequence. FBN, fixed bin number; FBW, fixed bin width

The median and interquartile (first to third) range ICC 95% CI lower bound values of radiomic feature extracted from both T1-weighted and T2-weighted sequences are reported in Table 6, grouped according to image intensity discretization method and segmentation approach. A significant difference among ICC values was found using Friedman test for repeated samples on both T1-weighted and T2-weighted sequences (*p* < 0.001). In pairwise comparisons, higher feature ICC 95% CI lower bound values were found when performing image intensity discretization with fixed bin width compared to fixed bin number, regardless of the segmentation approach, on both T1-weighted and T2-weighted images (*p* < 0.001). On T1-weighted images, no difference in terms of ICC 95% CI lower bound was found between contour-focused and margin shrinkage segmentations after both discretization methods with fixed bin number (*p* = 0.8) and width (*p* = 0.62). On T2-weighted images, no difference in terms of ICC 95% CI lower bound was found between the two segmentation approaches after discretization with fixed bin number (*p* = 0.24). On T2-weighted images, higher ICC 95% CI lower bound values were found when performing margin shrinkage segmentation after intensity discretization with fixed bin width, compared to contour-focused segmentation (*p* < 0.001). In Fig. 4, box and whisker plots show the interobserver reproducibility of all features extracted from each MRI sequence using different discretization methods and segmentation approaches.

**Table 6:** ICC values by discretization method and segmentation approach. Median and interquartile (first to third) range ICC 95% CI lower bound values of radiomic features extracted from both T1-weighted and T2-weighted sequences, grouped according to discretization method and segmentation approach

**Image**	**Discretization method**	**Segmentation approach**	**ICC 95% CI lower bound**	
			**Median**	**Interquartile range (**first to third**)**
**T1w**	**Fixed bin number**	**Contour focused**	0.971	0.932–0.986
		**Margin shrinkage**	0.974	0.929–0.986
	**Fixed bin width**	**Contour focused**	0.982	0.957–0.992
		**Margin shrinkage**	0.983	0.957–0.992
**T2w**	**Fixed bin number**	**Contour focused**	0.954	0.900–0.978
		**Margin shrinkage**	0.955	0.907–0.983
	**Fixed bin width**	**Contour focused**	0.969	0.936–0.989
		**Margin shrinkage**	0.977	0.939–0.991

## Discussion

The main finding of our study is that the rates of stable radiomic features extracted from T1-weighted and T2-weighted MRI sequences were very high (90% or higher) regardless of the discretization method and segmentation approach. The discretization method with fixed bin width yielded higher stable feature rates and higher feature ICC values compared to fixed bin number, regardless of the segmentation approach with or without marginal erosion (*p* < 0.001). Additionally, no difference in stable feature rates was found between the segmentation approaches, regardless of the discretization method (*p* ≥ 0.175). Overall, a small but still not negligible degree of segmentation variability highlighted the need to include a reliability analysis in radiomic studies.

Radiomics has a great potential as a non-invasive biomarker to quantify several tumor characteristics, both standalone and combined with artificial intelligence methods such as machine learning [31–33]. However, it faces challenges to clinical implementation [34]. A great variability in radiomic features has emerged as a major issue across studies, and image segmentation is the most critical step [11]. As segmentation is time-consuming if performed manually, prior to conducting radiomic studies, methodological analyses would be desirable to preliminarily evaluate the robustness of different segmentation approaches and avoid biases due to non-reproducible, noisy features. Similar analyses were previously performed in kidney [16], lung and head and neck [14], and cartilaginous bone [15] lesions. Regarding lipomatous soft-tissue tumors, most radiomic studies included a feature reproducibility assessment as a dimensionality-reduction method in their radiomic workflow, which was built with the aim of differentiating benign from malignant (including low-grade) lesions [35–42]. More recently, Sudjai et al. compared the effects of intra- and interobserver segmentation variability on the reproducibility of 2D and 3D MRI-based radiomic feature reproducibility in lipoma and ALT [43]. A region growing-based semiautomatic contour-focused segmentation was performed on T1-weighted sequences by two readers and only original images were used for feature extraction, resulting in 43 out of 93 (46.2%) 2D features and 76 out of 107 (71%) 3D features with an absolute agreement ICC ≥ 0.75, which defined feature stability [43]. Based on their findings, we focused our study on 3D segmentations only, as they yielded higher stable feature rates. We compared two image intensity discretization methods (fixed bin number vs. fixed bin width) and two segmentation approaches (contour-focused vs. margin shrinkage) on both T1-weighted and T2-weighted sequences, involving three different readers as suggested by the ICC guidelines by Koo and Li [24]. After extraction of features from original, filtered and transformed images (1106 features per sequence compared to 107 in the previous study [43]), we found higher rates of stable features (90% or higher per sequence, regardless of the discretization method and segmentation approach) using ICC 95% CI lower bound ≥ 0.75 as a stricter cutoff to define feature stability. This difference could be attributed to the use of filtered and transformed (in addition to the original) images for feature extraction in our study, as well as to the different experiences of the readers involved in image segmentation, namely a statistician and a research scientist in the previous study [43] and three physicians in our study. Despite these differences, a common conclusion that can be drawn from the previous [43] and our studies is that most 3D MRI radiomic features of lipoma and ALT have good reproducibility, although a certain degree of segmentation variability exists.

In our study, T1-weighted and T2-weighted MRI sequences demonstrated good reproducibility regardless of the image intensity discretization method employed in image pre-processing, which was performed using both options of fixed bin number and fixed bin width, with stable feature rates respectively ranging from 90.69 to 95.66% and from 95.39 to 97.65%. The discretization method with fixed bin width resulted in higher stable feature rates and higher feature ICC values, thus providing more robust features compared to discretization with fixed bin number in our series. This finding is in line with previous positron emission tomography and MRI studies showing better feature reproducibility when implementing fixed bin width [44, 45]. Margin shrinkage led to an improvement in terms of feature ICC values compared to contour-focused segmentation only when implementing discretization with fixed bin width on T2-weighted images. Conversely, no difference in terms of feature ICC values was found between the two segmentation approaches when implementing discretization with fixed bin width on T1-weighted images or fixed bin number regardless of the employed MRI sequence. Additionally, no difference in terms of stable feature rates was found between the two segmentation approaches, regardless of the discretization method. Thus, a definite conclusion regarding the superiority of one segmentation approach over the other cannot be drawn. This confirms the need for a preliminary assessment of feature reproducibility in radiomic workflows and is in line with literature emphasizing the importance of reproducibility in artificial intelligence and radiology [46–48].

Some limitations of our study should be addressed. First, it has a retrospective design, as a prospective analysis is not strictly necessary for radiomic studies [49]. Second, the retrospective design accounts for the exclusion of contrast-enhanced MRI, which was not performed consistently in our series of lipomas and ALTs. This is in line with recent studies suggesting that the value of contrast administration may be limited in lipoma and ALT [6, 50], with no clear improvement in diagnostic accuracy following the addition of contrast-enhanced sequences to a non-contrast MRI protocol [50]. Finally, due to its scope, this was a single institution study, and the generalizability of our results should be confirmed on more varied datasets.

## Conclusions

Radiomic features of lipoma and ALT extracted from T1-weighted and T2-weighted MRI sequences are reproducible regardless of the segmentation approach and segmentation method, although a minimal degree of segmentation variability exists and highlights the need to perform a preliminary reproducibility analysis in radiomic studies. As stable feature rates were similar between contour-focused and margin shrinkage segmentations, it could be reasonable to prefer the former approach for ease of use in clinical practice. Image intensity discretization with fixed bin width provided higher stable feature rates and feature ICC values compared to discretization with fixed bin number. Thus, the former discretization method might be favored when performing image pre-processing in future radiomic studies dealing with lipomatous soft-tissue tumors.

### Supplementary Information

Below is the link to the electronic supplementary material.

Supplementary file1 (XLSX 23 KB)

Supplementary file2 (XLSX 22 KB)

Supplementary file3 (XLSX 23 KB)

Supplementary file4 (XLSX 23 KB)

## Abbreviations

ALT: Atypical lipomatous tumor
CI: Confidence interval
GLCM: Gray-level cooccurrence matrix
GLDM: Gray-level dependence matrix
GLRLM: Gray-level run length matrix
GLSZM: Gray-level size zone matrix
ICC: Intraclass correlation coefficient
LoG: Laplacian of Gaussian
MRI: Magnetic resonance imaging
NGTDM: Neighboring gray tone difference matrix
ROI: Region of interest

## References

[1] Yee EJ, Stewart CL, Clay MR, McCarter MM (2022). Lipoma and Its Doppelganger. Surg Clin North Am.

[2] WHO Classification of Tumours Editorial Board (2020). WHO Classification of Tumours: Soft Tissue and Bone Tumours.

[3] Gronchi A, Miah AB, Dei Tos AP, Abecassis N, Bajpai J, Bauer S, Biagini R, Bielack S, Blay JY, Bolle S, Bonvalot S, Boukovinas I, Bovee JVMG, Boye K, Brennan B, Brodowicz T, Buonadonna A, De Álava E, Del Muro XG, Dufresne A, Eriksson M, Fagioli F, Fedenko A, Ferraresi V, Ferrari A, Frezza AM, Gasperoni S, Gelderblom H, Gouin F, Grignani G, Haas R, Hassan AB, Hecker-Nolting S, Hindi N, Hohenberger P, Joensuu H, Jones RL, Jungels C, Jutte P, Kager L, Kasper B, Kawai A, Kopeckova K, Krákorová DA, Le Cesne A, Le Grange F, Legius E, Leithner A, Lopez-Pousa A, Martin-Broto J, Merimsky O, Messiou C, Mir O, Montemurro M, Morland B, Morosi C, Palmerini E, Pantaleo MA, Piana R, Piperno-Neumann S, Reichardt P, Rutkowski P, Safwat AA, Sangalli C, Sbaraglia M, Scheipl S, Schöffski P, Sleijfer S, Strauss D, Strauss S, Sundby Hall K, Trama A, Unk M, van de Sande MAJ, van der Graaf WTA, van Houdt WJ, Frebourg T, Casali PG, Stacchiotti S (2021). Soft tissue and visceral sarcomas: ESMO–EURACAN–GENTURIS Clinical Practice Guidelines for diagnosis, treatment and follow-up. Ann Oncol.

[4] Wilson MP, Haidey J, Murad MH, Sept L, Low G: Diagnostic accuracy of CT and MR features for detecting atypical lipomatous tumors and malignant liposarcomas: a systematic review and meta-analysis. Eur Radiol, 10.1007/s00330-023-09916-2, July 13, 2023 10.1007/s00330-023-09916-2 37439933

[5] Knebel C, Neumann J, Schwaiger BJ, Karampinos DC, Pfeiffer D, Specht K, Lenze U, von Eisenhart-Rothe R, Rummeny EJ, Woertler K, Gersing AS (2019). Differentiating atypical lipomatous tumors from lipomas with magnetic resonance imaging: a comparison with MDM2 gene amplification status. BMC Cancer.

[6] Nardo L, Abdelhafez YG, Acquafredda F, Schirò S, Wong AL, Sarohia D, Maroldi R, Darrow MA, Guindani M, Lee S, Zhang M, Moawad AW, Elsayes KM, Badawi RD, Link TM (2020). Qualitative evaluation of MRI features of lipoma and atypical lipomatous tumor: results from a multicenter study. Skeletal Radiol.

[7] O’Donnell PW, Griffin AM, Eward WC, Sternheim A, White LM, Wunder JS, Ferguson PC (2013). Can Experienced Observers Differentiate between Lipoma and Well-Differentiated Liposarcoma Using Only MRI?. Sarcoma.

[8] Haidey J, Low G, Wilson MP (2023). Radiomics-based approaches outperform visual analysis for differentiating lipoma from atypical lipomatous tumors: a review. Skeletal Radiol.

[9] Gitto S, Cuocolo R, Annovazzi A, Anelli V, Acquasanta M, Cincotta A, Albano D, Chianca V, Ferraresi V, Messina C, Zoccali C, Armiraglio E, Parafioriti A, Sciuto R, Luzzati A, Biagini R, Imbriaco M, Sconfienza LM (2021). CT radiomics-based machine learning classification of atypical cartilaginous tumours and appendicular chondrosarcomas. EBioMedicine.

[10] Gitto S, Cuocolo R, van Langevelde K, van de Sande MAJ, Parafioriti A, Luzzati A, Imbriaco M, Sconfienza LM, Bloem JL (2022). MRI radiomics-based machine learning classification of atypical cartilaginous tumour and grade II chondrosarcoma of long bones. EBioMedicine.

[11] Gillies RJ, Kinahan PE, Hricak H (2016). Radiomics: Images Are More than Pictures. They Are Data. Radiology.

[12] Berenguer R (2018). Pastor-Juan M del R, Canales-Vázquez J, Castro-García M, Villas MV, Mansilla Legorburo F, Sabater S: Radiomics of CT Features May Be Nonreproducible and Redundant: Influence of CT Acquisition Parameters. Radiology.

[13] Gitto S, Corino VDA, Annovazzi A, Milazzo Machado E, Bologna M, Marzorati L, Albano D, Messina C, Serpi F, Anelli V, Ferraresi V, Zoccali C, Aliprandi A, Parafioriti A, Luzzati A, Biagini R, Mainardi L, Sconfienza LM: 3D vs. 2D MRI radiomics in skeletal Ewing sarcoma: Feature reproducibility and preliminary machine learning analysis on neoadjuvant chemotherapy response prediction. Front Oncol 12:1016123, 2022 10.3389/fonc.2022.1016123 PMC9755864 36531029

[14] Pavic M, Bogowicz M, Würms X, Glatz S, Finazzi T, Riesterer O, Roesch J, Rudofsky L, Friess M, Veit-Haibach P, Huellner M, Opitz I, Weder W, Frauenfelder T, Guckenberger M, Tanadini-Lang S (2018). Influence of inter-observer delineation variability on radiomics stability in different tumor sites. Acta Oncol.

[15] Gitto S, Cuocolo R, Emili I, Tofanelli L, Chianca V, Albano D, Messina C, Imbriaco M, Sconfienza LM (2021). Effects of Interobserver Variability on 2D and 3D CT- and MRI-Based Texture Feature Reproducibility of Cartilaginous Bone Tumors. J Digit Imaging.

[16] Kocak B, Ates E, Durmaz ES, Ulusan MB, Kilickesmez O (2019). Influence of segmentation margin on machine learning–based high-dimensional quantitative CT texture analysis: a reproducibility study on renal clear cell carcinomas. Eur Radiol.

[17] Duron L, Balvay D, Vande Perre S, Bouchouicha A, Savatovsky J, Sadik J-C, Thomassin-Naggara I, Fournier L, Lecler A (2019). Gray-level discretization impacts reproducible MRI radiomics texture features. PLoS One.

[18] Veres G, Vas NF, Lyngby Lassen M, Béresová M, K. Krizsan A, Forgács A, Berényi E, Balkay L: Effect of grey-level discretization on texture feature on different weighted MRI images of diverse disease groups. PLoS One 16:e0253419, 2021 10.1371/journal.pone.0253419 PMC8213143 34143830

[19] Schwier M, van Griethuysen J, Vangel MG, Pieper S, Peled S, Tempany C, Aerts HJWL, Kikinis R, Fennessy FM, Fedorov A (2019). Repeatability of Multiparametric Prostate MRI Radiomics Features. Sci Rep.

[20] Gitto S, Cuocolo R, Albano D, Morelli F, Pescatori LC, Messina C, Imbriaco M, Sconfienza LM (2021). CT and MRI radiomics of bone and soft-tissue sarcomas: a systematic review of reproducibility and validation strategies. Insights Imaging.

[21] Gitto S, Bologna M, Corino VDA, Emili I, Albano D, Messina C, Armiraglio E, Parafioriti A, Luzzati A, Mainardi L, Sconfienza LM (2022). Diffusion-weighted MRI radiomics of spine bone tumors: feature stability and machine learning-based classification performance. Radiol Med.

[22] Ugga L, Cuocolo R, Solari D, Guadagno E, D’Amico A, Somma T, Cappabianca P (2019). del Basso de Caro ML, Cavallo LM, Brunetti A: Prediction of high proliferative index in pituitary macroadenomas using MRI-based radiomics and machine learning. Neuroradiology.

[23] Zwanenburg A, Vallières M, Abdalah MA, Aerts HJWL, Andrearczyk V, Apte A, Ashrafinia S, Bakas S, Beukinga RJ, Boellaard R, Bogowicz M, Boldrini L, Buvat I, Cook GJR, Davatzikos C, Depeursinge A, Desseroit M-C, Dinapoli N, Dinh CV, Echegaray S, El Naqa I, Fedorov AY, Gatta R, Gillies RJ, Goh V, Götz M, Guckenberger M, Ha SM, Hatt M, Isensee F, Lambin P, Leger S, Leijenaar RTH, Lenkowicz J, Lippert F, Losnegård A, Maier-Hein KH, Morin O, Müller H, Napel S, Nioche C, Orlhac F, Pati S, Pfaehler EAG, Rahmim A, Rao AUK, Scherer J, Siddique MM, Sijtsema NM, Socarras Fernandez J, Spezi E, Steenbakkers RJHM, Tanadini-Lang S, Thorwarth D, Troost EGC, Upadhaya T, Valentini V, van Dijk LV, van Griethuysen J, van Velden FHP, Whybra P, Richter C, Löck S (2020). The Image Biomarker Standardization Initiative: Standardized Quantitative Radiomics for High-Throughput Image-based Phenotyping. Radiology.

[24] Koo TK, Li MY (2016). A Guideline of Selecting and Reporting Intraclass Correlation Coefficients for Reliability Research. J Chiropr Med.

[25] Yushkevich PA, Piven J, Hazlett HC, Smith RG, Ho S, Gee JC, Gerig G (2006). User-guided 3D active contour segmentation of anatomical structures: Significantly improved efficiency and reliability. Neuroimage.

[26] Jenkinson M, Beckmann CF, Behrens TEJ, Woolrich MW, Smith SM (2012). FSL. Neuroimage.

[27] van Griethuysen JJM, Fedorov A, Parmar C, Hosny A, Aucoin N, Narayan V, Beets-Tan RGH, Fillion-Robin J-C, Pieper S, Aerts HJWL (2017). Computational Radiomics System to Decode the Radiographic Phenotype. Cancer Res.

[28] Di Leo G, Sardanelli F: Statistical significance: p value, 0.05 threshold, and applications to radiomics—reasons for a conservative approach. Eur Radiol Exp 4:18, 2020 10.1186/s41747-020-0145-y PMC7064671 32157489

[29] van der Walt S, Colbert SC, Varoquaux G (2011). The NumPy Array: A Structure for Efficient Numerical Computation. Comput Sci Eng.

[30] R Core Team: R: A language and environment for statistical computing, 2020

[31] Fanciullo C, Gitto S, Carlicchi E, Albano D, Messina C, Sconfienza LM (2022). Radiomics of Musculoskeletal Sarcomas: A Narrative Review. J Imaging.

[32] Yin X, Liao H, Yun H, Lin N, Li S, Xiang Y, Ma X (2022). Artificial intelligence-based prediction of clinical outcome in immunotherapy and targeted therapy of lung cancer. Semin Cancer Biol.

[33] Pang J, Xiu W, Ma X (2023). Application of Artificial Intelligence in the Diagnosis, Treatment, and Prognostic Evaluation of Mediastinal Malignant Tumors. J Clin Med.

[34] Cuocolo R, Caruso M, Perillo T, Ugga L, Petretta M (2020). Machine Learning in oncology: A clinical appraisal. Cancer Lett.

[35] Cay N, Mendi BAR, Batur H, Erdogan F (2022). Discrimination of lipoma from atypical lipomatous tumor/well-differentiated liposarcoma using magnetic resonance imaging radiomics combined with machine learning. Jpn J Radiol.

[36] Foreman SC, Llorián-Salvador O, David DE, Rösner VKN, Rischewski JF, Feuerriegel GC, Kramp DW, Luiken I, Lohse A-K, Kiefer J, Mogler C, Knebel C, Jung M, Andrade-Navarro MA, Rost B, Combs SE, Makowski MR, Woertler K, Peeken JC, Gersing AS: Development and Evaluation of MR-Based Radiogenomic Models to Differentiate Atypical Lipomatous Tumors from Lipomas. Cancers (Basel) 15:2150, 2023 10.3390/cancers15072150 PMC10093205 37046811

[37] Gitto S, Interlenghi M, Cuocolo R, Salvatore C, Giannetta V, Badalyan J, Gallazzi E, Spinelli MS, Gallazzi M, Serpi F, Messina C, Albano D, Annovazzi A, Anelli V, Baldi J, Aliprandi A, Armiraglio E, Parafioriti A, Daolio PA, Luzzati A, Biagini R, Castiglioni I, Sconfienza LM (2023). MRI radiomics-based machine learning for classification of deep-seated lipoma and atypical lipomatous tumor of the extremities. Radiol Med.

[38] Leporq B, Bouhamama A, Pilleul F, Lame F, Bihane C, Sdika M, Blay J-Y, Beuf O (2020). MRI-based radiomics to predict lipomatous soft tissue tumors malignancy: a pilot study. Cancer Imaging.

[39] Malinauskaite I, Hofmeister J, Burgermeister S, Neroladaki A, Hamard M, Montet X, Boudabbous S (2020). Radiomics and Machine Learning Differentiate Soft-Tissue Lipoma and Liposarcoma Better than Musculoskeletal Radiologists. Sarcoma.

[40] Sudjai N, Siriwanarangsun P, Lektrakul N, Saiviroonporn P, Maungsomboon S, Phimolsarnti R, Asavamongkolkul A, Chandhanayingyong C (2023). Tumor-to-bone distance and radiomic features on MRI distinguish intramuscular lipomas from well-differentiated liposarcomas. J Orthop Surg Res.

[41] Tang Y, Cui J, Zhu J, Fan G (2022). Differentiation Between Lipomas and Atypical Lipomatous Tumors of the Extremities Using Radiomics. J Magn Reson Imaging.

[42] Yang Y, Zhou Y, Zhou C, Ma X (2022). Novel computer aided diagnostic models on multimodality medical images to differentiate well differentiated liposarcomas from lipomas approached by deep learning methods. Orphanet J Rare Dis.

[43] Sudjai N, Siriwanarangsun P, Lektrakul N, Saiviroonporn P, Maungsomboon S, Phimolsarnti R, Asavamongkolkul A, Chandhanayingyong C: Robustness of Radiomic Features: Two-Dimensional versus Three-Dimensional MRI-Based Feature Reproducibility in Lipomatous Soft-Tissue Tumors. Diagnostics (Basel) 13:258, 2023 10.3390/diagnostics13020258 PMC9858448 36673068

[44] Leijenaar RTH, Nalbantov G, Carvalho S, van Elmpt WJC, Troost EGC, Boellaard R, Aerts HJW., Gillies RJ, Lambin P: The effect of SUV discretization in quantitative FDG-PET Radiomics: the need for standardized methodology in tumor texture analysis. Sci Rep 5:11075, 2015 10.1038/srep11075 PMC4525145 26242464

[45] Koçak B, Yüzkan S, Mutlu S, Karagülle M, Kala A, Kadıoğlu M, Solak S, Sunman Ş, Temiz ZH, Ganiyusufoğlu AK: Influence of image preprocessing on the segmentation-based reproducibility of radiomic features: in vivo experiments on discretization and resampling parameters. Diagn Interv Radiol, 10.4274/dir.2023.232543, December 11, 2023 10.4274/dir.2023.232543 PMC11095065 38073244

[46] Akinci D’Antonoli T, Cavallo AU, Vernuccio F, Stanzione A, Klontzas ME, Cannella R, Ugga L, Baran A, Fanni SC, Petrash E, Ambrosini I, Cappellini LA, van Ooijen P, Kotter E, Pinto dos Santos D, Cuocolo R: Reproducibility of radiomics quality score: an intra- and inter-rater reliability study. Eur Radiol, 10.1007/s00330-023-10217-x, September 21, 2023 10.1007/s00330-023-10217-x PMC10957586 37733025

[47] Kocak B, Baessler B, Bakas S, Cuocolo R, Fedorov A, Maier-Hein L, Mercaldo N, Müller H, Orlhac F (2023). Pinto dos Santos D, Stanzione A, Ugga L, Zwanenburg A: CheckList for EvaluAtion of Radiomics research (CLEAR): a step-by-step reporting guideline for authors and reviewers endorsed by ESR and EuSoMII. Insights Imaging.

[48] Mongan J, Moy L, Kahn CE (2020). Checklist for Artificial Intelligence in Medical Imaging (CLAIM): A Guide for Authors and Reviewers. Radiol Artif Intell.

[49] Lubner MG, Smith AD, Sandrasegaran K, Sahani DV, Pickhardt PJ (2017). CT Texture Analysis: Definitions, Applications, Biologic Correlates, and Challenges. Radiographics.

[50] Shannon BA, Ahlawat S, Morris CD, Levin AS, Fayad LM (2022). Do contrast-enhanced and advanced MRI sequences improve diagnostic accuracy for indeterminate lipomatous tumors?. Radiol Med.

